# DecBin: Bayesian-optimized deep clustering for long-read metagenomic binning

**DOI:** 10.1093/bib/bbag571

**Published:** 2026-10-14

**Authors:** Yi-Heng Zhu, Xinchi Li, Zuo Liu, Jing Liu, Rui Ye, Wei Xue, Gaofei Jiang

**Affiliations:** College of Artificial Intelligence, Nanjing Agricultural University, 666 Binjiang Avenue, Jiangbei New District, Nanjing, Jiangsu 211800, China; College of Artificial Intelligence, Nanjing Agricultural University, 666 Binjiang Avenue, Jiangbei New District, Nanjing, Jiangsu 211800, China; College of Artificial Intelligence, Nanjing Agricultural University, 666 Binjiang Avenue, Jiangbei New District, Nanjing, Jiangsu 211800, China; College of Artificial Intelligence, Nanjing Agricultural University, 666 Binjiang Avenue, Jiangbei New District, Nanjing, Jiangsu 211800, China; College of Artificial Intelligence, Nanjing Agricultural University, 666 Binjiang Avenue, Jiangbei New District, Nanjing, Jiangsu 211800, China; College of Artificial Intelligence, Nanjing Agricultural University, 666 Binjiang Avenue, Jiangbei New District, Nanjing, Jiangsu 211800, China; Jiangsu Provincial Key Lab for Organic-based Fertilizer Creation and Soil Health Manipulation, Jiangsu Collaborative Innovation Center of Solid Organic Wastes, Educational Ministry Engineering Center of Resource-Saving Fertilizers, National Engineering Research Center for Organic-Based Fertilizers, College of Resources and Environmental Sciences, Nanjing Agricultural University, 666 Binjiang Avenue, Jiangbei New District, Nanjing, Jiangsu, 210095, China

**Keywords:** long reads, metagenomic binning, variational autoencoder, Bayesian optimization, deep clustering, joint fine-tuning

## Abstract

Accurate metagenomic binning is essential for reconstructing microbial genomes, thereby facilitating the discovery of novel taxa, the identification of pathogens, and the characterization of antimicrobial resistance genes. Most existing methods are designed for assembled contigs and cannot be directly applied to raw long reads. Although a few dedicated long-read binning methods have emerged, challenges remain in bin-number estimation and representation learning. Here, we propose Deep Embedded Clustering for Long-read Binning (DecBin), an adaptive method for directly binning raw long reads through integrating a variational autoencoder (VAE), Bayesian optimization, and deep embedded clustering within a unified framework. Computational experiments on large-scale simulated and mock-community datasets demonstrate that DecBin outperforms state-of-the-art methods in both long-read binning and metagenome-assembled genome recovery, while also achieving strain-level resolution in a low-complexity natural microbiome. Further analysis shows that the advantage of DecBin is partly attributable to its Davies–Bouldin index-guided Bayesian optimization, which explicitly evaluates candidate cluster numbers using a global cluster-validity criterion, thereby improving bin-number estimation across communities of varying complexity. Meanwhile, joint fine-tuning of the VAE encoder and clustering layer enables clustering feedback to refine the latent space, yielding representations better aligned with genome partitions. These findings highlight the potential of adaptive deep clustering for metagenomic binning directly from raw long reads without prior assembly. The source code of DecBin is freely available at https://github.com/LorMeBioAI/DecBin.

## Introduction

Metagenomic binning groups sequence fragments inferred to originate from the same genome into distinct bins, facilitating the reconstruction of microbial draft genomes [1–4]. Accurate binning is critical for improving genome completeness and reducing contamination, thereby supporting the discovery of novel microbial taxa, the identification of pathogenic microorganisms, and the characterization of antimicrobial resistance genes [5–8].

Conventional metagenomic binning is generally performed on contigs assembled from sequencing reads and relies on the assumption that sufficiently long contigs originating from the same genome exhibit similar sequence composition and abundance profiles [9–12]. For example, the Variational Autoencoder for Metagenomic Binning (VAMB) [13] uses a variational autoencoder (VAE) to learn latent representations from the composition and abundance features of contigs, followed by clustering with an iterative medoid algorithm. Adversarial Autoencoders (AAEs) for Metagenomic Binning [14] extends VAMB by employing an AAE to improve feature representation and binning accuracy (ACC). SemiBin [15] leverages deep contrastive learning with Siamese neural networks to incorporate must-link and cannot-link constraints between contigs. BASALT [16] integrates multiple assemblies and uses tetranucleotide frequency and the coverage correlation coefficient to improve binning ACC and the completeness of recovered genomes. LorBin [17] integrates VAE-based feature embedding, two-stage multiscale adaptive clustering, and bin-quality-guided reclustering to improve the recovery of high-quality genomes from imbalanced microbial communities.

Despite these advances in contig-level binning, the growing use of third-generation sequencing has created an increasing demand for methods that can directly bin raw long reads [18–21]. Third-generation sequencing technologies generate reads reaching lengths of up to 1 MB, thereby preserving richer genomic context and making direct binning prior to assembly increasingly feasible [18, 22–25]. However, existing contig-based methods cannot be directly applied to raw long reads because they generally rely heavily on coverage profiles estimated by mapping reads to assembled contigs, whereas reliable coverage profiles are difficult to obtain for individual long reads [26]. Moreover, sequencing errors and large differences in coverage among species can hinder the assembly of long and accurate contigs, particularly for low-abundance species, leading to fragmented assemblies and incomplete genome recovery [27].

Together, these challenges have motivated the development of dedicated methods for directly binning raw long reads prior to assembly. To date, only a limited number of methods have been developed for this purpose, with MetaBCC-LR [28] and LRBinner [26] being representative examples. MetaBCC-LR sequentially applies dimensionality reduction and density-based clustering to k-mer coverage histograms and oligonucleotide composition profiles. LRBinner uses a VAE to jointly encode compositional and abundance-related features, followed by independent clustering of the learned latent representations. Despite their effectiveness, both methods have two limitations. First, the number of output bins emerges from local density- or distance-based clustering rules, without explicitly comparing and optimizing candidate cluster numbers using a global cluster-validity metric. This may lead to inaccurate estimation of the number of genome bins, particularly in complex or imbalanced microbial communities. Second, feature representation and clustering are performed sequentially rather than being jointly optimized. Consequently, clustering feedback cannot be used to adapt the feature space, potentially compromising accurate read-to-genome assignment.

Motivated by these methodological limitations, we developed Deep Embedded Clustering for Long-read Binning (DecBin), an adaptive long-read metagenomic binning method through integrating the VAE, Bayesian optimization, and deep embedded clustering into a unified framework. Specifically, DecBin (i) learns informative latent representations directly from raw long reads using a VAE; (ii) uses Bayesian optimization guided by the Davies–Bouldin index (DBI) [29], a global cluster-validity metric, to adaptively estimate the number of clusters for initializing the deep clustering layer; and (iii) jointly fine-tunes the VAE encoder and deep clustering layer to refine latent representations and cluster assignments. Together, these designs contribute to improved long-read binning ACC and read utilization, thereby facilitating bin-specific assembly and metagenome-assembled genome (MAG) recovery. We demonstrate that DecBin consistently outperforms existing long-read binning approaches on both simulated and experimental mock-community datasets and achieves strain-level resolution in a low-complexity natural microbiome. These advances arise from two main algorithmic contributions: (a) DBI-guided Bayesian optimization for explicit bin-number estimation and (b) clustering-guided refinement of the latent space to better align learned representations with genome partitions.

## Materials and methods

### Datasets

Three types of datasets were selected to provide complementary evaluations of DecBin under simulated, experimental mock-community, and strain-level resolution settings.

#### Simulated datasets

Three labeled long-read metagenomic datasets, designated sim10, sim30, and sim70, were generated from reference genomes representing 10, 30, and 70 microbial species, respectively, using PBSIM2, a long-read sequencing simulator [30], in sampling-based mode with profiles derived from PacBio HiFi reads. For each dataset, the count of reads, sequencing depth, species composition and relative abundance, and genomic relatedness among the represented species are summarized in Supplementary Table S1 of the Supporting Information (SI). The known read-to-genome assignments enable direct quantitative evaluation of DecBin’s clustering performance across microbial communities differing in size, abundance structure, and genomic relatedness.

#### Experimental mock-community dataset

Three experimentally generated PacBio HiFi mock-community datasets, SRR13128012, SRR13128013, and SRR13128014, were obtained from the LRBinner study [26], comprising 21 strains from 17 species, with further composition details summarized in Supplementary Table S2. These datasets contain authentic sequencing noise and uneven species-abundance distributions, enabling evaluation of DecBin’s practical performance on real-world metagenomic data.

#### Strain-level resolution dataset

The natural whey starter microbiome dataset NWC2 [31] was specifically selected to evaluate DecBin’s ability to achieve strain-level resolution. This dataset mainly contains four bacterial strains from three species (see Supplementary Table S3 for composition details), including two genomically closely related *Lactobacillus helveticus* (*L. helveticus*) strains (NWC_2_3 and NWC_2_4), thereby providing a stringent test of intraspecific strain separation.

### Overview of the DecBin workflow

We developed DecBin, an adaptive long-read binning method that integrates the VAE [32], Bayesian optimization [33], and deep clustering, with the overall framework illustrated in Fig. 1. The method comprises five key components:

**Figure 1:**
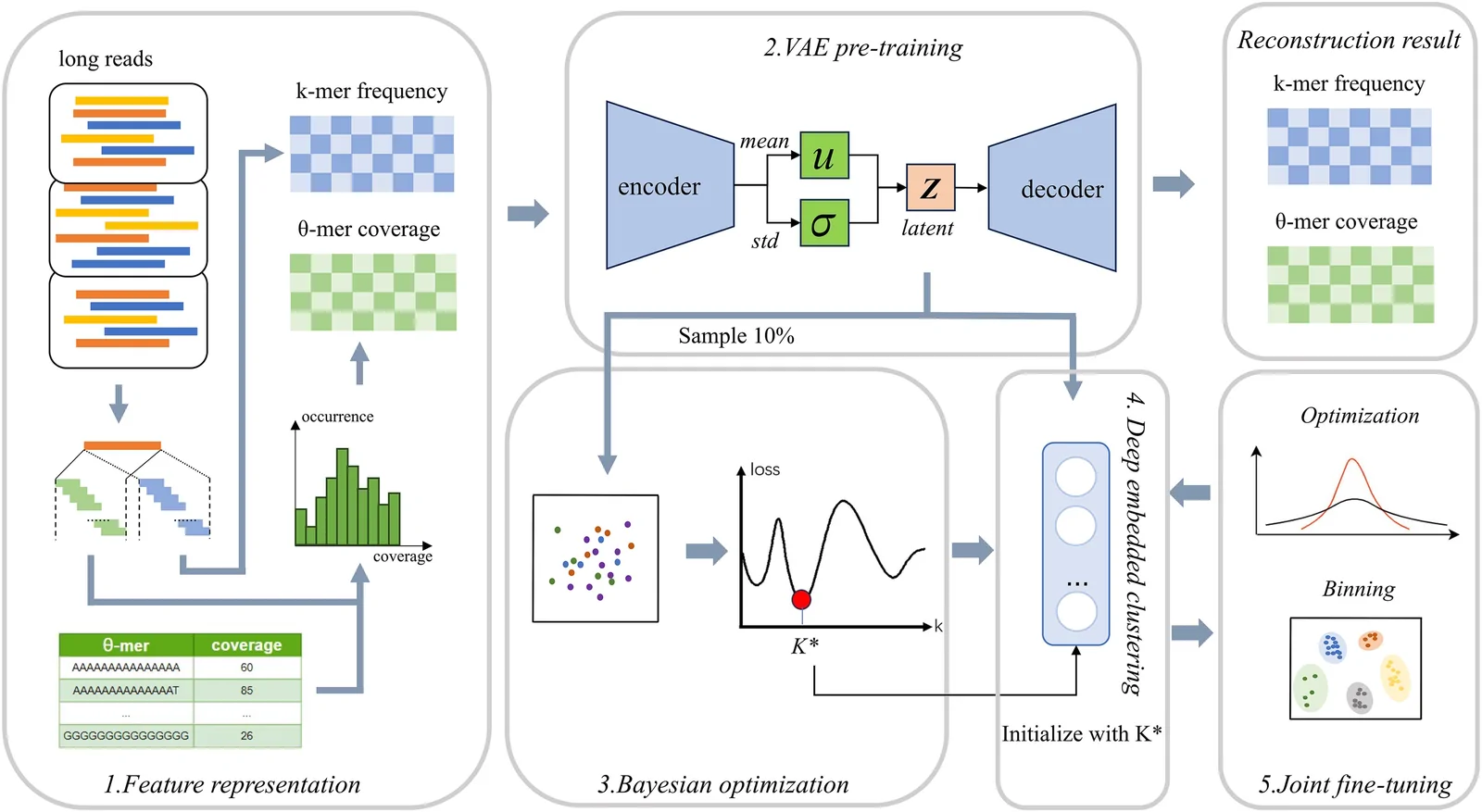
The framework of DecBin; (1) feature representation; (2) VAE pre-training; (3) Bayesian optimization; (4) deep embedded clustering; (5) joint fine-tuning.

**(1) Feature representation:** For each input metagenomic dataset, the *k*-mer frequency vector and coverage histogram of each long read were computed and concatenated to form its input feature vector.

**(2) VAE pre-training:** The resulting feature vectors from all long reads were used to pre-train the VAE in an unsupervised manner, producing a low-dimensional latent representation for each read.

**(3) Bayesian optimization:** A random 10% subset of the latent representations was used to iteratively evaluate candidate cluster numbers by applying *K*-means [34] with the DBI [29] as the objective function. The candidate yielding the minimum DBI was selected as the optimal number of clusters (${K}^{\ast }$).

**(4) Deep embedded clustering:** A clustering layer was appended to the VAE encoder and initialized using the ${K}^{\ast }$ cluster centers obtained by *K*-means. Student’s t-distribution-based similarity kernel was then used to convert the distances between each latent representation and the cluster centers into soft cluster-assignment probabilities.

**(5) Joint fine-tuning:** The VAE encoder and clustering layer were jointly fine-tuned to refine the latent representations and cluster assignments, after which each long read was assigned to a genome bin.

#### Feature representation

For each long read, two complementary alignment-free features were derived from its sequence, including a *k*-mer frequency vector [35] and a *θ*-mer coverage histogram vector [26].

To capture sequence composition, all *k*-mers in each read were extracted and counted using a sliding window of length *k* and a step size of 1. Each *k*-mer and its reverse complement were treated as the same category, yielding $T={4}^k/2$ categories of *k*-mers for odd *k* and $T=\left({4}^k+{4}^{k/2}\right)/2$ categories for even *k*, respectively. For the *i**-*th read, the occurrence counts of these *T* categories were collected into a raw count vector ${v}_i\in{\mathbb{R}}^T$, which was then normalized by the total count according to Equation (1) to produce the *k*-mer frequency vector ${x}_i^c\in{\mathbb{R}}^T$. The normalized vectors of all *N* reads in the input metagenomic dataset were stacked to form the *k*-mer frequency matrix ${X}^c\in{\mathbb{R}}^{N\times T}$.


(1) \begin{eqnarray*} {x}_{i,j}^c=\frac{v_{i,j}+{10}^{-5}}{\sum_{t=1}^T\left({v}_{i,t}+{10}^{-5}\right)} \end{eqnarray*}

To characterize abundance-related patterns, a *θ*-mer coverage histogram was constructed for each long read. First, the occurrence count of each *θ*-mer was calculated across the entire metagenomic dataset to construct a global coverage table, with *θ* chosen to be substantially larger than *k* so that the resulting *θ*-mers were more genome-specific [36]. For the *i**-*th read, all *θ*-mers were extracted using a sliding window of length *θ* and a step size of 1, and their corresponding dataset-wide counts were retrieved from the coverage table. These counts were grouped into *S* predefined intervals, and the resulting histogram was normalized by the total number of *θ*-mers in the read [analogous to Equation (1)] to obtain the coverage histogram vector ${x}_i^a\in{\mathbb{R}}^S$. The vectors of all *N* reads were stacked to form the *θ*-mer coverage histogram matrix ${X}^a\in{\mathbb{R}}^{N\times S}$.

#### Variational autoencoder pre-training

The coverage and composition feature matrices were concatenated as the input feature matrix $X=[{X}^a\Vert{X}^c]\in{\mathbb{R}}^{N\times \left(S+T\right)}$, which was then fed to the VAE to generate low-dimensional latent representations.

The encoder of the VAE compressed the input features through a two-layer fully connected network, with each layer followed by LeakyReLU activation [37], dropout, and batch normalization. For each read *i*, the encoder produced a mean vector ${u}_i\in{\mathbb{R}}^D$ and an standard deviation (SD) vector ${\sigma}_i\in{\mathbb{R}}^D$, from which a latent vector ${z}_i={u}_i+{\sigma}_i\bigodot{\varepsilon}_i\in{\mathbb{R}}^D$ was generated via the reparameterization trick. Here, ${\varepsilon}_i\in{\mathbb{R}}^D$ is a random noise vector sampled from the standard multivariate normal distribution $\mathbb{N}\left(0,\mathrm{I}\right)$, and *⨀* denotes element-wise multiplication. These latent vectors were then fed into the decoder, symmetric to the encoder, to reconstruct the input features, which were subsequently partitioned into the reconstructed coverage feature matrix ${X}_o^a$ and composition feature matrix ${X}_o^c$.

The loss function consists of the weighted reconstruction loss ${L}_R$ and Kullback–Leibler (KL) divergence loss ${L}_{KL}$, which are defined in Equations (2) and (3).


(2) \begin{eqnarray*} {L}_R=\frac{1}{2}{w}_1{\left\Vert{X}_o^a-{X}^a\right\Vert}^2+\frac{1}{2}{w}_2{\left\Vert{X}_o^c-{X}^c\right\Vert}^2 \end{eqnarray*}

(3) \begin{eqnarray*} {L}_{KL}=-\frac{1}{2}{w}_3\sum_{i=1}^N\sum_{j=1}^D\left(1+\log{\sigma_{i,j}}^2-{u}_{i,j}^2-{\sigma_{i,j}}^2\right) \end{eqnarray*}

Here, ${w}_1$, ${w}_2$, and ${w}_3$ are weighting coefficients. The VAE parameters were optimized using the Adam optimizer [38] by minimizing ${L}_R+{L}_{KL}$. During pre-training, the sampled vectors ${z}_i$ were used for reconstruction. After pre-training, the posterior means ${u}_i$ were used as the latent representations for subsequent clustering, thereby avoiding the sampling variability introduced by noise ${\varepsilon}_i$.

#### Bayesian optimization

We used Bayesian optimization to determine the optimal number of clusters for the subsequent deep clustering layer. To reduce computational cost, a randomly selected 10% subset of the latent representations generated by the pre-trained VAE was used during optimization. For each candidate cluster number *K*, *K*-means clustering [34] was performed on this subset, and the resulting clustering was then evaluated using the DBI [29]. Accordingly, the objective function *J(K)* was defined as shown in Equation (4).


(4) \begin{eqnarray*} J(K)= DBI(K)=\frac{1}{K}\sum_{i=1}^K\underset{1\le j\le K,j\ne i}{\max}\left(\frac{s_i+{s}_j}{d_{ij}}\right) \end{eqnarray*}

where ${s}_i$ is the mean distance of samples in cluster ${C}_i$ from its centroid ${c}_i$, ${s}_j$ is defined analogously for cluster ${C}_j$, and ${d}_{ij}$ is the distance between centroids ${c}_i$ and ${c}_j$. Lower DBI values indicate better clustering performance, characterized by more compact and better-separated clusters.

Given a search interval $\left[{K}_{\mathrm{down}},{K}_{\mathrm{up}}\right]$, an initial set of candidate cluster numbers was randomly selected and evaluated to obtain the corresponding objective values *J(K)*. The observed pairs $\left\{K,\kern0.5em J(K)\right\}$ were then used to guide the selection of the next unevaluated candidate. Specifically, the expected improvement (EI) acquisition function [39] ${EI}_{J^{\ast }}(K)$ was defined to quantify the expected reduction in the objective value relative to the lowest value observed so far, as shown in Equation (5).


(5) \begin{eqnarray*} {EI}_{J^{\ast }}(K)={\int}_{-\infty}^{+\infty}\max \left({J}^{\ast }-J,0\right)P\left(J|K\right) dJ \end{eqnarray*}

where ${J}^{\ast }$ represents the lowest objective value observed so far, and $P\left(J\mid K\right)$ is the probability density of the possible objective value *J* conditioned on the candidate cluster number *K*. The next candidate cluster number was selected by maximizing EI, as shown in Equation (6).


(6) \begin{eqnarray*} {K}_{new}=\underset{K\in \left[{K}_{\mathrm{down}},{K}_{\mathrm{up}}\right]}{\mathrm{argmax}}{EI}_{J^{\ast }}(K) \end{eqnarray*}

The EI-guided optimization was implemented using the tree-structured Parzen estimator (TPE) [40] as follows: (i) the TPE used the accumulated observations ${\mathcal{D}}_t={\left\{{K}_i,\kern0.5em J\left({K}_i\right)\right\}}_{i=1}^t$ to select the next candidate cluster number ${K}_{\mathrm{new}}$; (ii) *K*-means clustering was then performed with ${K}_{\mathrm{new}}$ clusters, and the corresponding $J\left({K}_{\mathrm{new}}\right)$ was calculated; and (iii) the resulting observation $\left({K}_{\mathrm{new}},J\left({K}_{\mathrm{new}}\right)\right)$ was added to ${\mathcal{D}}_t$ to generate ${\mathcal{D}}_{t+1}$ for the next iteration. This procedure was repeated until the predefined evaluation budget was reached. Finally, the optimal cluster number ${K}^{\ast }$ was selected from all evaluated candidates, as shown in Equation (7).


(7) \begin{eqnarray*} {K}^{\ast }=\underset{K\in{\mathcal{K}}_{\mathrm{eval}}}{\mathrm{argmin}}J(K) \end{eqnarray*}

where ${\mathcal{K}}_{\mathrm{eval}}$ denotes the set of candidate cluster numbers evaluated during optimization.

#### Deep embedded clustering

A clustering layer containing ${K}^{\ast }$ trainable cluster centers was appended to the pre-trained VAE encoder to form the deep embedded clustering model, where ${K}^{\ast }$ was determined by Bayesian optimization. The cluster centers were initialized using the centroids generated by employing *K*-means clustering with ${K}^{\ast }$ clusters on the latent representations generated by the pre-trained encoder for all reads in the input metagenomic dataset.

For each long read *i*, the clustering layer converted its latent representation ${u}_i$ (see details in the VAE pre-training section) into soft assignment probabilities using a Student’s t-distribution-based similarity kernel [41], as shown in Equation (8).


(8) \begin{eqnarray*} {q}_{ij}=\frac{{\left(1+\frac{{\left|\left|{u}_i-{c}_j\right|\right|}_2^2}{\alpha}\right)}^{-\frac{\alpha +1}{2}}}{\sum_{j^{\prime }=1}^{K^{\ast }}{\left(1+\frac{{\left|\left|{u}_i-{c}_{j^{\prime }}\right|\right|}_2^2}{\alpha}\right)}^{-\frac{\alpha +1}{2}}} \end{eqnarray*}

where ${q}_{ij}$ represents the probability of assigning read *i* to cluster *j*, ${c}_j$ represents the trainable center of cluster *j*, and *α* is the degrees-of-freedom parameter. The notation $\parallel \cdotp{\parallel}_2$ denotes the ${L}_2$ norm; thus, $\parallel{u}_i-{c}_j{\parallel}_2^2$ represents the squared Euclidean distance between the latent representation ${u}_i$ and the cluster center ${c}_j$.

#### Joint fine-tuning

To refine ambiguous soft assignments, an auxiliary target distribution $P=\left[{p}_{ij}\right]$ was constructed from the soft assignment distribution $Q=\left[{q}_{ij}\right]$, as shown in Equations (9) and (10).


(9) \begin{eqnarray*} {p}_{ij}=\frac{\frac{{q_{ij}}^2}{f_j}}{\sum_{j^{\prime }=1}^{K^{\ast }}\frac{{q_{i{j}^{\prime}}}^2}{f_{j^{\prime }}}} \end{eqnarray*}

(10) \begin{eqnarray*} {f}_j=\sum_{i=1}^N{q}_{ij} \end{eqnarray*}

where ${f}_j$ denotes the soft size of cluster *j*, calculated as the sum of the assignment probabilities of all reads to cluster *j*; squaring ${q}_{ij}$ strengthens confident assignments, whereas division by ${f}_j$ reduces the influence of differences in cluster sizes.

The pre-trained VAE encoder and the trainable cluster centers were then jointly fine-tuned using stochastic gradient descent [42] by minimizing the Kullback–Leibler divergence [43] between *P* and *Q*, as defined in Equation (11).


(11) \begin{eqnarray*} {L}_C={D}_{KL}\left(P\mid |Q\right)=\sum_{i=1}^N\sum_{j=1}^{K^{\ast }}{p}_{ij}\log \frac{p_{ij}}{q_{ij}} \end{eqnarray*}

After fine-tuning, each read was assigned to the cluster with the highest soft assignment probability.

### Evaluation metrics

To evaluate the clustering performance of DecBin, we used three complementary metrics, namely, accuracy (ACC), normalized mutual information (NMI), and adjusted Rand index (ARI), as defined in Zelig *et al*. [44]. ACC measures the proportion of correctly classified samples after matching the predicted cluster labels to the ground-truth class labels. NMI quantifies the amount of shared information between the clustering results and the true labels. ARI measures the pairwise agreement between the clustering results and the true groupings while correcting for agreement expected by chance.

To assess the quality of the bins obtained by each binning tool, we used CheckM [45] to determine the completeness and contamination of the bins. We defined high-quality bins as those with a completeness of ≥90% and a contamination of ≤5%; medium-quality bins as those with a completeness of ≥50% and a contamination of <10%; and low-quality bins as those with a completeness of <50% and a contamination of <10%.

### Implementation details and parameter settings

The hyperparameters for the individual algorithmic modules in DecBin were set as follows. In the feature representation module, the compositional *k*-mer size was set to *k=4* after benchmarking DecBin with *k=*3, 4, 5, and 6 across three simulated datasets, with the corresponding ACC values reported in Supplementary Table S4 of SI. Overall, *k=4* achieved the highest average ACC while maintaining lower computational and storage costs. Values greater than 6 were not further evaluated because of the rapidly increasing computational cost. For *k=4*, reverse-complementary *k*-mers were merged, resulting in $T=\left({4}^k+{4}^{k/2}\right)/2=136$ compositional categories. Following the parameter setting used in previous work [26], the *θ*-mer size used to construct the coverage histogram was set to *θ =15*, while coverage values were grouped into *S=32* intervals spanning 1–320, with an interval width of 10.

In the VAE module, the encoder comprised two fully connected hidden layers with 128 neurons each and a dropout rate of 0.1, mapping the input features into a (*D=32*) latent space. The loss weights were set to ${w}_1=0.1$ for coverage reconstruction, ${w}_2=1$ for composition reconstruction, and ${w}_3=0.01$ for KL divergence.

In the Bayesian optimization module, the search bounds were set to ${K}_{\mathrm{down}}=0.5{n}_{\mathrm{sp}}$ and ${K}_{\mathrm{up}}=1.5{n}_{\mathrm{sp}}$, where ${n}_{\mathrm{sp}}$ denotes an approximate species number used to initialize a computationally tractable candidate range. Bayesian optimization subsequently selected the optimal cluster number ${K}^{\ast }$ within this range. For the simulated datasets, the predefined species number was used as ${n}_{\mathrm{sp}}$. For the experimental mock-community datasets, where the species number was not known in advance, ${n}_{\mathrm{sp}}$ was estimated from species-level classifications generated by Kraken2 [46], with further details provided in Supplementary Text S1.

DecBin was implemented in Python 3.9 using PyTorch 1.11.0 + cu113 and trained with a learning rate of $1\times{10}^{-3}$, a batch size of 1024, and a maximum of 100 training epochs. This method was run independently five times without fixing the random seed, with 10% of the latent representations randomly sampled without replacement in each run for Bayesian optimization.

All experiments were conducted under Ubuntu 20.04.1 Long Term Support (LTS) on a workstation equipped with a 32-core/64-thread central processing unit (CPU), 64 GB of system memory, and an NVIDIA GeForce RTX 4090 graphics processing unit (GPU) with 24 GB of memory. Feature extraction, including *k*-mer composition and *θ*-mer coverage calculation, was performed using 16 CPU threads, whereas model training was performed using a single CPU thread with GPU acceleration.

## Results

### Benchmark experiments on simulated datasets

We benchmarked DecBin against two state-of-the-art long-read binning methods, MetaBCC-LR [28] and LRBinner [26], on three simulated datasets (sim10, sim30, sim70). The overall performance of the three methods is compared in Fig. 2a and b, while the numbers of recovered bins and the corresponding ACC values are reported in Tables 1 and 2, respectively. Moreover, the computational costs of the three methods, measured by runtime and peak memory usage, are further compared in Supplementary Table S5 of SI. The reported runtime represents the total wall-clock time for a complete run, including both feature extraction and model training.

**Figure 2:**
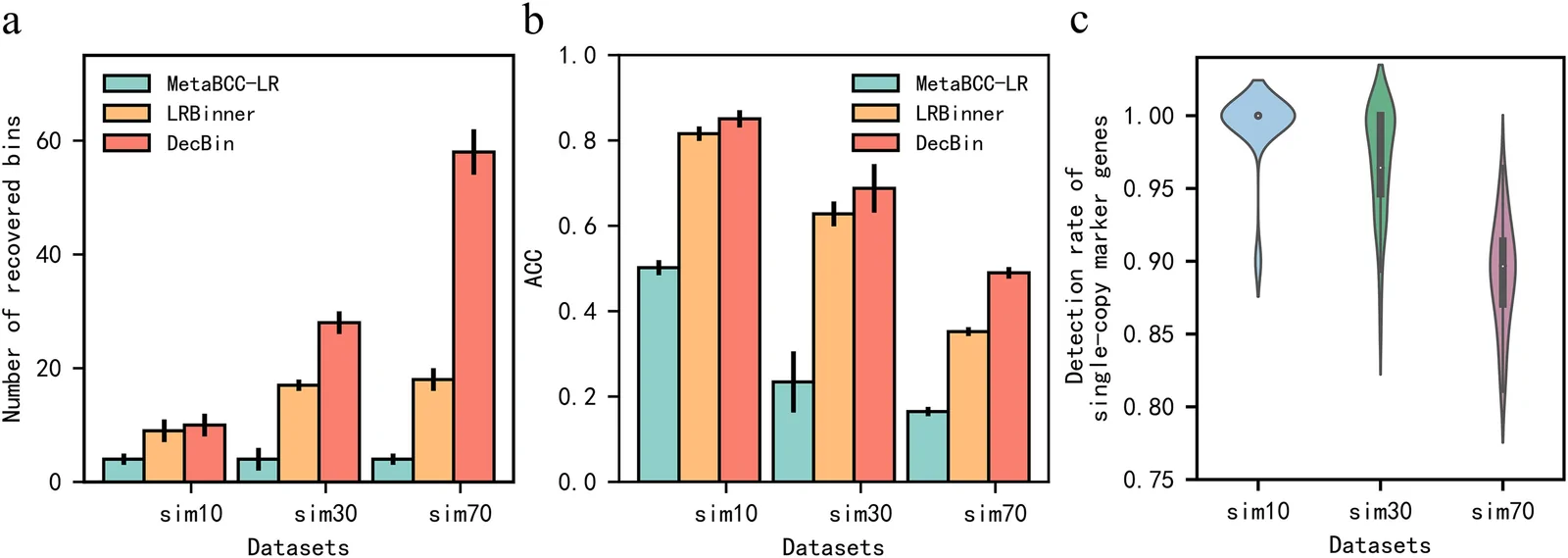
Comparison of MetaBCC-LR, LRBinner, and DecBin on three simulated datasets; (a) number of recovered bins; (b) ACC values; (c) distributions of the detection rates of 43 single-copy marker genes across the bins recovered by DecBin.

**Table 1:** Number of clusters identified by MetaBCC-LR, LRBinner, and DecBin on three simulated datasets.

Method	sim10	sim30	sim70
MetaBCC-LR	4 ± 1	4 ± 2	4 ± 1
LRBinner	9 ± 2	17 ± 1	18 ± 2
DecBin	**10 ± 2**	**28 ± 2**	**58 ± 4**

Note: Values are reported as mean ± SD across repeated runs. Bold values indicate the results closest to the true number of genomes for each dataset.

**Table 2:** ACC values of MetaBCC-LR, LRBinner, and DecBin on three simulated datasets.

Method	sim10	sim30	sim70
MetaBCC-LR	0.5019 ± 0.0278	0.2342 ± 0.0719	0.1648 ± 0.0110
LRBinner	0.8158 ± 0.0170	0.6279 ± 0.0293	0.3521 ± 0.0105
DecBin	**0.8506 ± 0.0204**	**0.6878 ± 0.0570**	**0.4898 ± 0.0137**

Note: Values are reported as mean ± SD across repeated runs. Bold values indicate the best-performing result.

Overall, DecBin achieved substantially better clustering performance than the two competing methods, with only a moderate increase in runtime and memory usage. Specifically, DecBin used Bayesian optimization to identify clusters that most closely match the true number of genomes and yielded 10, 28, and 58 clusters for sim10, sim30, and sim70, respectively. In comparison, LRBinner identified 9, 17, and 18 clusters for the three datasets, whereas MetaBCC-LR found only 4 clusters across all datasets. DecBin also achieved the highest ACC values of 0.8506, 0.6878, and 0.4898 for sim10, sim30, and sim70, respectively, and surpassed the second-best method, LRBinner, by 4.3%, 9.5%, and 39.1%, respectively.

To further evaluate the recovery of conserved core genomic regions, we used MetaCHIP [47] to identify 43 single-copy marker genes and examined their distribution across the bins recovered by DecBin. For each marker gene, the detection rate was calculated as the proportion of recovered bins in which it was detected. Figure 2c summarizes the distributions of marker-gene detection rates across the three simulated datasets, while the detection rates of individual marker genes are shown in Supplementary Figs S1, S2, and S3. Detection rates ranged from 90.0% to 100% for sim10, 85.7% to 100% for sim30, and 81.0% to 96.6% for sim70. Despite a moderate decline with increasing community complexity, marker-gene detection remained consistently high across the DecBin-recovered bins. These results suggest that DecBin generally preserves conserved core genomic regions across communities of varying complexity.

Given the potential impact of repetitive regions on read assignment, we further assessed DecBin’s robustness by separately evaluating reads identified by RepeatMasker [48] as originating from repetitive genomic regions. DecBin achieved ACC values of 0.8274, 0.7058, and 0.4979 for these reads on sim10, sim30, and sim70, respectively. These results closely matched the corresponding overall ACC values, with differences of no more than 2.32 percentage points, suggesting that DecBin is relatively robust to repetitive genomic regions. Together, these analyses suggest that DecBin exhibits robust performance across conserved and repetitive genomic regions.

### Contribution analysis of key DecBin components

To systematically investigate the contributions of DecBin’s key algorithmic components, we conducted component-level analyses on the three simulated datasets. Specifically, we evaluated the effects of (1) Bayesian optimization for cluster-number estimation and (2) joint fine-tuning of the VAE encoder and clustering layer.

(1) Bayesian optimization improves cluster-number estimation

To evaluate the contribution of Bayesian optimization to cluster-number estimation, we replaced it with random search while keeping the upstream components unchanged, including feature representation and VAE pre-training. For both strategies, the same *K*-means clustering procedure, search range, number of candidate evaluations, and DBI objective were used. As shown in Supplementary Table S6 of the SI, both strategies estimated 10 clusters for the sim10 dataset, matching the expected species number of 10. For sim30 and sim70, random search estimated 23 and 50 clusters, respectively, whereas Bayesian optimization estimated 28 and 58 clusters, which were closer to the expected species numbers of 30 and 70. These results indicate that Bayesian optimization provides more accurate cluster-number estimation than random search, particularly for more complex microbial communities.

(2) Joint fine-tuning enhances binning performance

DecBin uses the deep clustering layer to bin the fine-tuned features. The clustering assignments obtained during initialization were used as the starting point for fine-tuning. We evaluated the effects of the clustering layer on ACC, NMI, and ARI during fine-tuning across three simulated datasets, as illustrated in Fig. 3a. All three metrics improved by at least 1%. The curves of ACC, NMI, and ARI indicated that the clustering layer brought features from long reads within the same cluster closer together while increasing the separation between different clusters.

**Figure 3:**
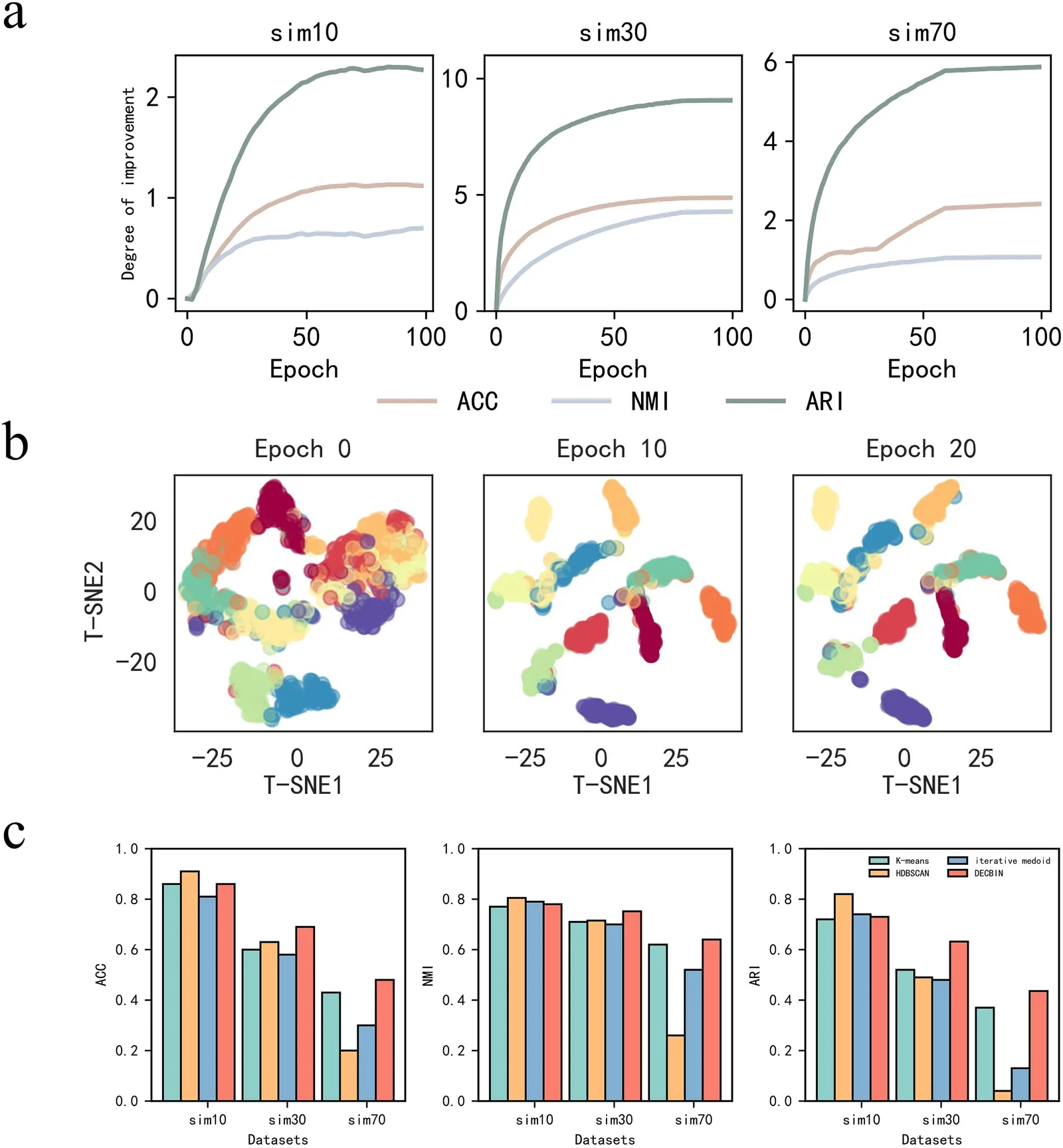
Joint fine-tuning of the VAE encoder and clustering layer enhances binning performance; (a) improvements in ACC, NMI, and ARI during fine-tuning on simulated datasets; (b) t-distributed stochastic neighbor embedding visualization of the latent embeddings of 1000 long reads sampled from the sim10 dataset at epochs 0, 10, and 20, with colors representing species labels; (c) comparison of ACC, NMI, and ARI between DecBin and three baseline algorithms that were applied to fixed feature embeddings without joint fine-tuning of the VAE encoder and clustering.

To demonstrate how joint training of the VAE and clustering layers reshaped the latent feature space, we sampled 1000 long reads from the sim10 dataset and visualized their latent embeddings via t-distributed stochastic neighbor embedding [49], as shown in Fig. 3b. As joint training progressed, the boundaries between different clusters became increasingly distinct. Long reads from the same species moved closer together, whereas those from different species became more widely separated.

To further assess the benefits of jointly optimizing feature learning by the VAE encoder and clustering, we conducted ablation experiments on the three simulated datasets using *K*-means, a simplified Hierarchical Density-Based Spatial Clustering of Applications with Noise (HDBSCAN) procedure from MetaBCC-LR, and iterative medoid clustering from LRBinner. These three algorithms were applied to fixed feature embeddings generated by the pre-trained VAE encoder and did not jointly optimize feature learning and cluster assignments. We compared the ACC, NMI, and ARI values between DecBin and these three methods, as summarized in Fig. 3c. DecBin achieved the best overall performance on sim30 and sim70. On sim30, DecBin achieved 0.6878 (ACC), 0.7517 (NMI), and 0.6319 (ARI), surpassing the best-performing method without joint optimization of feature learning and clustering by at least 5% across all three evaluation metrics. On sim70, DecBin achieved 0.4898 (ACC), 0.6478 (NMI), and 0.4358 (ARI), outperforming the best competing method by at least 2%. HDBSCAN performed best on sim10, which contained the fewest species. However, its performance declined markedly as species diversity increased, suggesting that HDBSCAN is better suited to less complex datasets with fewer species.

Overall, these results demonstrate that jointly optimizing feature learning and clustering improves the quality of the learned embeddings and enhances clustering performance, particularly on datasets with greater species diversity and complexity.

### Performance on experimental mock-community datasets

We further benchmarked DecBin against two competing methods, MetaBCC-LR and LRBinner, on three experimental mock-community datasets (SRR13128012, SRR13128013, and SRR13128014). Specifically, the long reads assigned to each bin by each method were assembled separately using metaFlye [50], and the resulting bin-specific contigs were collectively treated as a candidate MAG and assessed for completeness and contamination using CheckM [45]. Additionally, to assess bin purity, reads within each bin were aligned against the corresponding reference genomes using minimap2 [51]. Following the definition of genome-bin purity used in the Critical Assessment of Metagenome Interpretation: the second round of challenges [21], purity was calculated as the proportion of base pairs assigned to the dominant reference genome among all reference-assigned base pairs within each bin. The numbers of medium- and high-quality MAGs (hmMAGs) recovered by the three binning methods are summarized in Fig. 4a. The number of high-quality MAGs and their mean completeness, contamination, and purity across the three datasets are summarized in Table 3, with statistical differences in the three quality metrics between DecBin and each competing method assessed using a two-sided Mann–Whitney U test [52] followed by Benjamini–Hochberg correction for multiple comparisons [53]. Detailed results for individual MAGs are reported in Supplementary Table S7 of SI.

**Figure 4:**
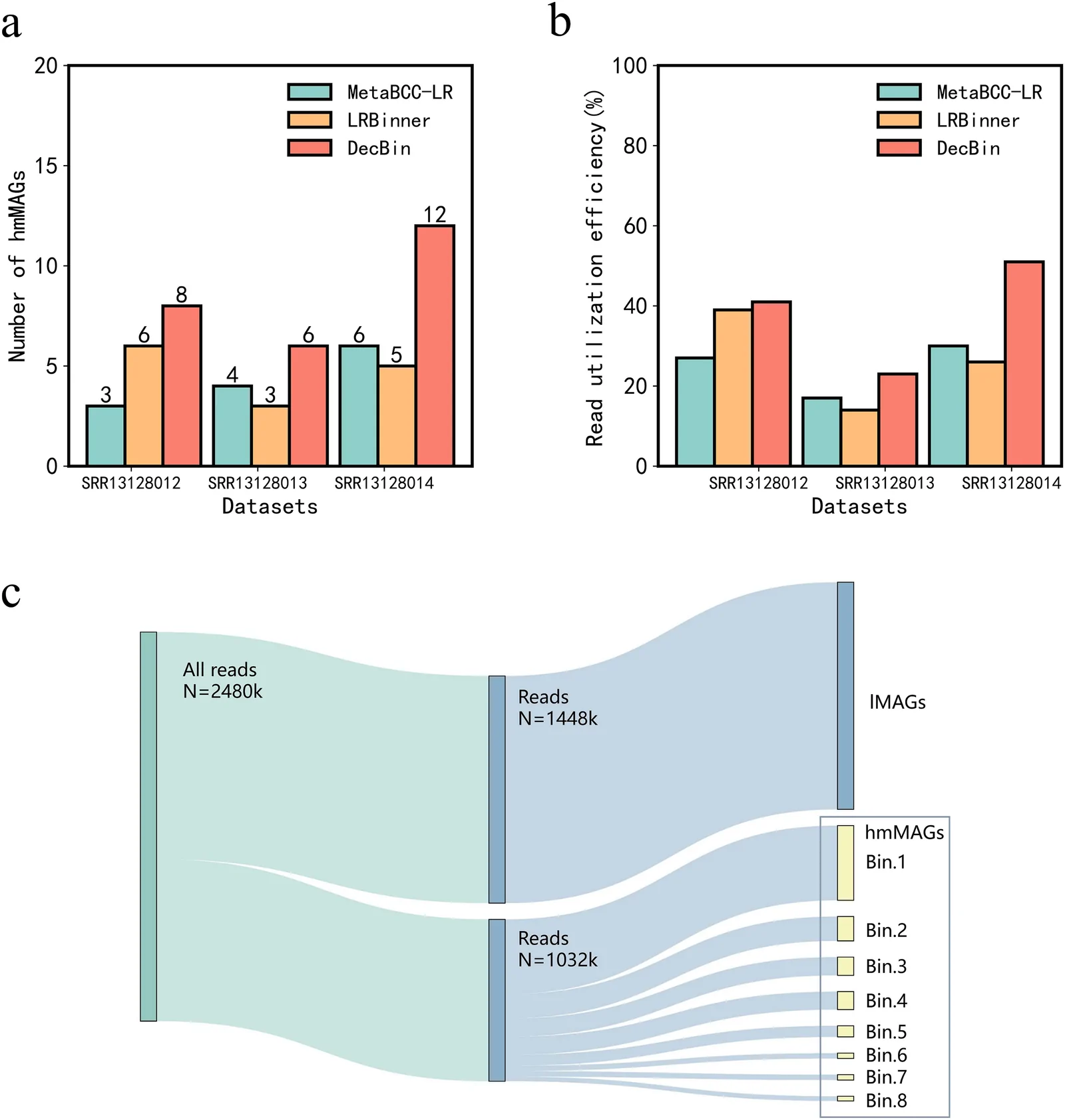
Comparison of DecBin with two competing binners on experimental mock-community datasets; (a) number of hmMAGs recovered by the three binners; (b) read utilization efficiency, calculated as the percentage of raw reads contributing to the hmMAGs; (c) Sankey diagram illustrating the complete binning workflow of DecBin on the SRR13128012 dataset; all the reads were partitioned into distinct bins, with 41.6% contributing to hmMAGs and the remainder contributing to low-quality MAGs (lMAGs).

**Table 3:** Number and quality metrics of high-quality MAGs recovered by three binning methods across three experimental mock-community datasets.

Metric	DecBin	LRBinner	MetaBCC-LR
Number of high-quality MAGs	18	13	10
Mean completeness (%)	98.76	99.70 (*P* = .224)	98.94 (*P* = .248)
Mean contamination (%)	1.15	0.42 (*P* = .224)	0.23 (*P* = .147)
Mean purity (%)	98.85	98.49 (*P* = .271)	99.58 (*P* = .092)

*P*-values in the LRBinner and MetaBCC-LR columns indicate comparisons with DecBin using a two-sided Mann–Whitney U test with Benjamini–Hochberg correction for multiple comparisons.

Overall, DecBin recovered more hmMAGs than the two competing methods across the three experimental mock-community datasets. Specifically, it recovered 8, 6, and 12 hmMAGs from SRR13128012, SRR13128013, and SRR13128014, respectively, yielding 26 in total, compared with 14 for LRBinner and 13 for MetaBCC-LR. Among these, 18 were high-quality MAGs, compared with 13 for LRBinner and 10 for MetaBCC-LR (Table 3). The advantage in high-quality MAG recovery was particularly evident on SRR13128013 and SRR13128014, with DecBin outperforming both competing methods (Supplementary Table S7).

Beyond recovery counts, high-quality MAGs recovered by DecBin achieved mean completeness, contamination, and purity of 98.76%, 1.15%, and 98.85%, respectively (Table 3). None of these metrics differed significantly between DecBin and either LRBinner or MetaBCC-LR (all *P* > .05). For medium-quality MAGs, DecBin gained corresponding values of 87.13%, 3.91%, and 98.59%, respectively. Because only a limited number of medium-quality MAGs were recovered by the competing methods, particularly LRBinner with only one medium-quality MAG, these values were interpreted descriptively without formal statistical testing. Notably, DecBin recovered seven MAGs with 100% completeness across the three datasets, exceeding the corresponding totals of three for LRBinner and four for MetaBCC-LR (Supplementary Table S7). These results indicate that DecBin recovered more high- and medium-quality MAGs while maintaining high completeness and purity with controlled contamination, suggesting that its advantage is primarily reflected in broader genome recovery.

The read utilization efficiencies of the three methods, calculated as the percentage of raw reads contributing to the hmMAGs, are presented in Fig. 4b. DecBin achieved read utilization efficiencies of 41.6%, 23.5%, and 51.6% on the three datasets, respectively, consistently exceeding those of the two competing methods. This indicates that a larger proportion of the raw reads contributed to bins that ultimately yielded hmMAGs, supporting the broader genome recovery achieved by DecBin. Additionally, to illustrate how reads were partitioned and subsequently contributed to MAGs of different quality categories, we visualized the flow of reads through the DecBin pipeline for the SRR13128012 dataset using a Sankey diagram, as shown in Fig. 4c.

### Strain-level resolution in a natural whey starter culture

To evaluate DecBin’s ability to distinguish closely related strains, we analyzed the NWC2 natural whey starter microbiome, a low-complexity community dominated by four bacterial strains representing three species, with its detailed composition summarized in Supplementary Table S3. To quantify their genomic relatedness, we calculated pairwise average nucleotide identity (ANI) among the four reference genomes using FastANI [54], with the resulting ANI matrix shown in Fig. 5a. This analysis showed that the two *L. helveticus* strains, NWC_2_3 and NWC_2_4, shared an ANI of 98.94%, whereas all other pairwise ANI values were below 77%. Therefore, the high genomic similarity between NWC_2_3 and NWC_2_4 makes distinguishing these two strains particularly challenging and provides a stringent test of strain-level resolution.

**Figure 5:**
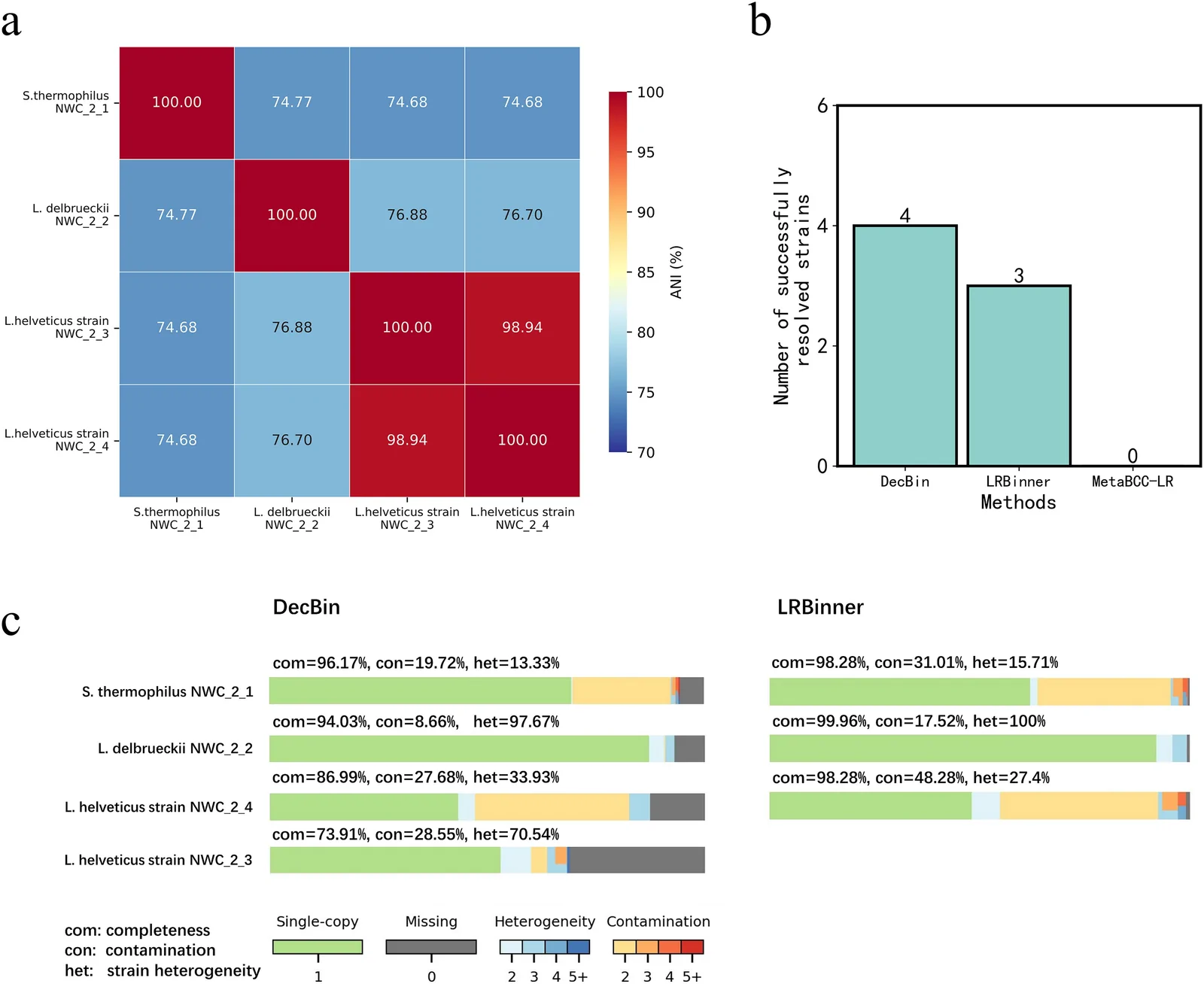
Strain-level resolution of three binning methods on the NWC2 natural whey starter microbiome; (a) ANI matrix of the four reference genomes, calculated using FastANI; to facilitate comparisons among relatively divergent genomes, FastANI was run with relaxed parameters (−−fragLen = 1000 bp and –−minFraction = 0.05; defaults: 3000 bp and 0.20), allowing ANI estimates below 80%; the –−fragLen parameter defines the fragment length used for mapping, whereas –−minFraction defines the minimum fraction of successfully mapped fragments required for ANI estimation; because FastANI is primarily validated within the 80%–100% ANI range, ANI estimates below 80% obtained under these relaxed settings should be interpreted with caution; (b) number of successfully resolved strains; (c) CheckM marker-gene profiles of the strain-associated assemblies recovered by DecBin and LRBinner, together with their estimated completeness, contamination, and strain heterogeneity.

We further compared DecBin with two competing binning methods, LRBinner and MetaBCC-LR, on the NWC2 dataset. For each method, reads assigned to individual bins were assembled separately using metaFlye [50], and the resulting contigs were aligned to the four reference genomes using minimap2 [51]. A strain was considered successfully resolved when the contigs from a distinct bin could be assigned to its reference genome. The completeness and contamination of each strain-associated assembly were subsequently assessed using CheckM [45].

Figure 5b summarizes the number of strains successfully resolved by each method, while Fig. 5c presents the CheckM marker-gene profiles of the strain-associated assemblies recovered by DecBin and LRBinner, together with their completeness, contamination, and strain heterogeneity values. Overall, DecBin showed the strongest strain recovery performance, successfully resolving all four strains, compared with three resolved by LRBinner and 0 by MetaBCC-LR. The four strains resolved by DecBin achieved completeness values of 73.91%–96.17%, contamination values of 8.66%–28.55%, and strain heterogeneity values of 13.33%–97.67%. For the three strains resolved by both DecBin and LRBinner, DecBin consistently yielded substantially lower contamination, despite slightly lower completeness. The high strain heterogeneity observed for *Lactobacillus delbrueckii* (*L. delbrueckii*) NWC_2_2 and *L. helveticus* NWC_2_3 indicates that part of the contamination originated from closely related strains. Notably, DecBin was the only method that resolved both highly similar *L. helveticus* strains, demonstrating its stronger strain-level resolution on the NWC2 dataset.

## Discussion and conclusion

In this work, we proposed DecBin, an adaptive method for long-read metagenomic binning that integrates the VAE, Bayesian optimization, and deep embedded clustering into a unified framework. DecBin was systematically benchmarked against state-of-the-art methods on both simulated and experimental mock-community datasets, demonstrating superior binning performance and strain-level resolution, thereby facilitating metagenomic genome reconstruction. This performance advantage can be attributed to the following algorithmic innovations, which partly address the limitations of existing methods.

First, DecBin enables explicit, validity-guided estimation of the cluster number by using Bayesian optimization with the DBI, rather than relying on cluster numbers that emerge indirectly from the local density- or distance-based clustering rules used by existing methods. This explicit optimization strategy improves cluster-number estimation across communities of varying complexity. Second, DecBin jointly fine-tunes the VAE encoder and the deep embedded clustering layer, rather than separating representation learning from clustering as in previous methods, thereby enabling the simultaneous optimization of latent representations and cluster assignments. This joint optimization enhances intra-cluster compactness and inter-cluster separation, yielding representations better aligned with genome partitions. Moreover, the consistent performance improvements shown in Fig. 3c, particularly on the more complex sim30 and sim70 datasets, indicate that clustering-aware representation refinement may be especially beneficial for communities with greater diversity and complexity.

These findings suggest that the two key components of DecBin, namely, (i) DBI-guided Bayesian optimization for cluster-number estimation and (ii) joint optimization of representation learning and clustering, may inform future improvements to existing long-read binning frameworks. Explicit, validity-guided cluster-number estimation could reduce reliance on heuristic local clustering rules, while clustering feedback may yield latent representations better aligned with genome partitions. However, their transferability and effectiveness across different architectures require further evaluation.

Despite these advances, DecBin has several limitations. First, Bayesian optimization and joint fine-tuning introduce additional computational overhead. Second, its current feature representation does not leverage pretrained genomic language models [55], which may further improve feature representation and binning performance. Work in these directions is ongoing.

**Key Points**

- DecBin is an adaptive long-read metagenomic binning method integrating the variational autoencoder, Bayesian optimization, and deep embedded clustering into a unified framework.
- Davies–Bouldin index-guided Bayesian optimization automatically infers bin numbers, avoiding limitations of fixed or manual cluster number settings.
- Joint optimization of representation learning and clustering generates embeddings better aligned with biological genome partitions.
- DecBin outperforms existing tools in genome recovery and strain-level resolution across both simulated and experimental mock-community datasets.

## Supplementary Material

supplementary_bbag57

## Data Availability

Three PacBio HiFi mock-community datasets, SRR13128012-SRR13128014, were obtained from the National Center for Biotechnology Information (NCBI) (https://www.ncbi.nlm.nih.gov) under BioProject number PRJNA680590. NWC2 reads were obtained from the NCBI BioSample SAMN09580370 under the SRA accession codes SRX4451758 (Nanopore) and SRX4451757 (PacBio). The source code of DecBin is freely available at https://github.com/LorMeBioAI/DecBin.
