# Supplementary material for: DecBin: Bayesian-optimized deep clustering for long-read metagenomic binning
Source: Brief Bioinform. 2026 Oct 14;27(5):bbag571. doi: 10.1093/bib/bbag571 (PMC13662220; doi:10.1093/bib/bbag571)
Supplement: Supplementary file 1 [file supplementary_bbag57.docx]

**Supporting Information**

**DecBin: Bayesian-optimized deep clustering for**

**long-read metagenomic binning**

Yi-Heng Zhu^1,#,^ Xinchi Li^1,#^, Zuo Liu^1^, Jing Liu^1^, Rui Ye^1^, Wei Xue^1,*^ and Gaofei Jiang^2,*^

^1^ College of Artificial Intelligence, Nanjing Agricultural University, Nanjing, Jiangsu, 211800, China;

^2^ Jiangsu Provincial Key Lab for Organic-based Fertilizer Creation and Soil Health Manipulation, Jiangsu Collaborative Innovation Center of Solid Organic Wastes, Educational Ministry Engineering Center of Resource-Saving Fertilizers, National Engineering Research Center for Organic-Based Fertilizers, College of Resources and Environmental Sciences, Nanjing Agricultural University, Nanjing, Jiangsu, 210095, China

^#^ These authors contributed equally to this work.

*Corresponding authors. Wei Xue, College of Artificial Intelligence, Nanjing Agricultural University, Nanjing, Jiangsu, 211800, China. E-mail: xwsky@njau.edu.cn; Gaofei Jiang, Jiangsu Provincial Key Lab for Organic-based Fertilizer Creation and Soil Health Manipulation, Jiangsu Collaborative Innovation Center of Solid Organic Wastes, Educational Ministry Engineering Center of Resource-Saving Fertilizers, National Engineering Research Center for Organic-Based Fertilizers, College of Resources and Environmental Sciences, Nanjing Agricultural University, Nanjing, Jiangsu, 210095, China. E-mail: gjiang@njau.edu.cn

**Table of contents**

**Supporting Text**

- Text S1. Determination of the cluster-number search range for Bayesian optimization

**Supporting Figures**

- Figure S1. Single-copy marker-gene detection rates on the sim10 dataset.
- Figure S2. Single-copy marker-gene detection rates on the sim30 dataset.
- Figure S3. Single-copy marker-gene detection rates on the sim70 dataset.

**Supporting Tables**

- Table S1. Composition details of the three simulated datasets
- Table S2. Composition details of the three experimental mock-community datasets.
- Table S3. Composition details of the strain-level resolution dataset.
- Table S4. Performance of DecBin with different k-mer sizes on three simulated datasets.
- Table S5. Runtime and peak memory usage of MetaBCC-LR, LRBinner, and DecBin on three simulated datasets.
- Table S6. Cluster-number estimation by random search and Bayesian optimization on three simulated datasets
- Table S7. Completeness, contamination, and purity of high- and medium-quality MAGs recovered by DecBin, LRBinner, and MetaBCC-LR from three experimental mock-community datasets.

**Text S1. Determination of the cluster-number search range for Bayesian optimization**

In the Bayesian optimization module, the search bounds were set to $K_{\mathrm{down}}=0.5n_{\mathrm{sp}}$ and $K_{\mathrm{up}}=1.5n_{\mathrm{sp}}$, where $n_{\mathrm{sp}}$ denotes an approximate species number used to initialize a computationally tractable candidate range. Bayesian optimization subsequently selected the final cluster number $K^{*}$ within this range.

For the simulated datasets, the predefined species number was used as $n_{\mathrm{sp}}$ to establish standardized search ranges for controlled benchmarking. This setting was used for benchmark evaluation and does not imply that the true species number is required in practical applications. For the experimental mock-community datasets, where the species number was not known in advance, $n_{\mathrm{sp}}$ was estimated from the input reads using Kraken2 [1]. Specifically, species-level assignments were ranked according to the number of classified reads, and the read counts were accumulated until at least 80% of the species-classified reads were covered. The number of species required to reach this threshold was used as $n_{\mathrm{sp}}$. This procedure estimates the number of predominant and potentially recoverable species rather than the total species richness of the sample.

It should be noted that the search interval $[0.5n_{\mathrm{sp}}, 1.5n_{\mathrm{sp}}]$ used in this study was adopted as a computationally efficient setting for benchmark validation rather than as a universal constraint for practical applications. The interval width may be adjusted according to sample complexity and available computational resources. To provide empirical context for the plausible scale of $n_{\mathrm{sp}}$ in practical applications, we conducted a statistical analysis of publicly available long-read metagenomic datasets.

We searched the NCBI Sequence Read Archive (SRA) using “metagenome” as the search term and restricted the results to single-end long-read datasets generated using Oxford Nanopore Technologies (ONT) or Pacific Biosciences (PacBio) platforms. After excluding samples smaller than 1 GB, 529 eligible samples were retained for analysis. For each sample, Kraken2 was used to obtain species-level assignments, and the number of predominant species was estimated using the same 80% cumulative-abundance criterion. The estimated number of predominant species ranged from 5 to 468, with a mean of approximately 105, a standard deviation of approximately 92, and a median of 71; approximately 60% of the samples contained no more than 100 predominant species.

**Table S1. Composition details of the three simulated datasets**

| Dataset | Count of Reads | Total Size | Species | Sequence depth ^a^ | Relative abundance (%) ^b^ | Max genomic relatedness ^c^  (%) |
| --- | --- | --- | --- | --- | --- | --- |
| sim10 | 466680 | 2.0GB | Alistipes finegoldii | 80 | 14.23 | NA ^d^ |
|  |  |  | Anaerobutyricum hallii | 50 | 8.37 | NA |
|  |  |  | Bifidobacterium adolescentis | 25 | 2.49 | NA |
|  |  |  | Clostridium hylemonae | 60 | 10.98 | NA |
|  |  |  | Eubacterium rectale | 25 | 4.10 | NA |
|  |  |  | Gemella morbillorum | 200 | 16.93 | NA |
|  |  |  | Paraprevotella xylaniphila | 30 | 5.88 | NA |
|  |  |  | Ruminococcus gnavus | 150 | 25.35 | NA |
|  |  |  | Streptococcus mutans | 70 | 6.62 | NA |
|  |  |  | Veillonella parvula | 50 | 5.06 | NA |
| sim30 | 647983 | 2.8GB | Alistipes finegoldii | 60 | 7.67 | 85.84 |
|  |  |  | Alistipes shahii | 25 | 3.27 | 85.84 |
|  |  |  | Anaerobutyricum hallii | 50 | 6.03 | NA |
|  |  |  | Bacteroides caccae | 25 | 3.02 | NA |
|  |  |  | Bacteroides intestinalis | 15 | 2.98 | NA |
|  |  |  | Bifidobacterium adolescentis | 30 | 2.16 | 82.93 |
|  |  |  | Bifidobacterium angulatum | 20 | 1.42 | 82.93 |
|  |  |  | Bifidobacterium animalis | 25 | 1.67 | 79.44 |
|  |  |  | Bifidobacterium bifidum | 20 | 1.50 | 82.18 |
|  |  |  | Bifidobacterium breve | 25 | 1.99 | 88.19 |
|  |  |  | Bifidobacterium longum | 10 | 0.78 | 88.19 |
|  |  |  | Blautia producta | 10 | 2.18 | NA |
|  |  |  | Clostridium botulinum | 20 | 2.65 | NA |
|  |  |  | Clostridium hylemonae | 60 | 7.88 | NA |
|  |  |  | Clostridium saccharolyticum | 15 | 2.39 | NA |
|  |  |  | Eggerthella lenta | 25 | 3.08 | NA |
|  |  |  | Eubacterium rectale | 40 | 4.74 | NA |
|  |  |  | Fusobacterium ulcerans | 20 | 2.43 | NA |
|  |  |  | Gemella morbillorum | 125 | 7.64 | NA |
|  |  |  | Isoptericola variabilis | 30 | 3.41 | NA |
|  |  |  | Lactobacillus crispatus | 25 | 2.01 | NA |
|  |  |  | Lautropia mirabilis | 10 | 1.08 | NA |
|  |  |  | Methanosphaera stadtmanae | 15 | 0.91 | NA |
|  |  |  | Paraprevotella xylaniphila | 40 | 5.65 | NA |
|  |  |  | Phascolarctobacterium succinatutens | 30 | 2.41 | NA |
|  |  |  | Porphyromonas asaccharolytica | 10 | 0.75 | NA |
|  |  |  | Ruminococcus gnavus | 80 | 9.74 | NA |
|  |  |  | Streptococcus agalactiae | 10 | 0.74 | NA |
|  |  |  | Streptococcus mutans | 60 | 4.09 | NA |
|  |  |  | Veillonella parvula | 50 | 3.73 | NA |
| sim70 | 857913 | 3.7GB | Acidaminococcus intestini | 10 | 0.60 | NA |
|  |  |  | Adlercreutzia equolifaciens | 5 | 0.38 | 79.81 |
|  |  |  | Akkermansia muciniphila | 5 | 0.35 | NA |
|  |  |  | Alistipes finegoldii | 15 | 1.60 | 85.84 |
|  |  |  | Alistipes shahii | 30 | 2.96 | 85.84 |
|  |  |  | Anaerobutyricum hallii | 30 | 2.73 | NA |
|  |  |  | Bacteroides caccae | 20 | 2.61 | NA |
|  |  |  | Bacteroides intestinalis | 20 | 3.00 | NA |
|  |  |  | Bifidobacterium adolescentis | 50 | 2.85 | 84.35 |
|  |  |  | Bifidobacterium angulatum | 5 | 0.27 | 82.93 |
|  |  |  | Bifidobacterium bifidum | 20 | 1.14 | 82.18 |
|  |  |  | Bifidobacterium breve | 15 | 0.88 | 88.19 |
|  |  |  | Bifidobacterium longum | 20 | 1.29 | 88.19 |
|  |  |  | Bifidobacterium pseudocatenulatum | 10 | 0.60 | 84.35 |
|  |  |  | Blautia producta | 30 | 4.85 | NA |
|  |  |  | Campylobacter concisus | 30 | 1.42 | NA |
|  |  |  | Campylobacter hominis | 20 | 0.88 | 79.84 |
|  |  |  | Campylobacter ureolyticus | 10 | 0.47 | 79.84 |
|  |  |  | Clostridioides difficile | 15 | 1.57 | NA |
|  |  |  | Clostridium botulinum | 5 | 0.51 | NA |
|  |  |  | Clostridium hylemonae | 10 | 1.02 | 78.64 |
|  |  |  | Clostridium saccharolyticum | 30 | 3.62 | NA |
|  |  |  | Clostridium scindens | 15 | 1.42 | 78.64 |
|  |  |  | Collinsella aerofaciens | 30 | 1.76 | NA |
|  |  |  | Desulfovibrio piger | 40 | 2.90 | NA |
|  |  |  | Eggerthella lenta | 10 | 0.91 | 79.81 |
|  |  |  | Eubacterium eligens | 40 | 2.21 | NA |
|  |  |  | Faecalitalea cylindroides | 30 | 0.25 | NA |
|  |  |  | Finegoldia magna | 40 | 1.85 | NA |
|  |  |  | Fusobacterium mortiferum | 10 | 0.70 | 78.84 |
|  |  |  | Fusobacterium necrophorum | 15 | 1.04 | NA |
|  |  |  | Fusobacterium nucleatum | 15 | 0.70 | 77.92 |
|  |  |  | Fusobacterium ulcerans | 10 | 0.92 | 78.84 |
|  |  |  | Gardnerella vaginalis | 150 | 6.67 | NA |
|  |  |  | Gemella haemolysans | 10 | 0.50 | 81.25 |
|  |  |  | Gemella morbillorum | 15 | 0.69 | 81.25 |
|  |  |  | Haemophilus pittmaniae | 5 | 0.28 | NA |
|  |  |  | Helicobacter pylori | 10 | 0.42 | NA |
|  |  |  | Hungatella hathewayi | 10 | 1.80 | NA |
|  |  |  | Isoptericola variabilis | 10 | 0.86 | NA |
|  |  |  | Lactobacillus crispatus | 20 | 1.22 | 79.27 |
|  |  |  | Lactobacillus jensenii | 20 | 0.84 | 79.27 |
|  |  |  | Lactococcus lactis | 30 | 1.86 | NA |
|  |  |  | Leptotrichia wadei | 50 | 3.13 | NA |
|  |  |  | Megasphaera elsdenii | 5 | 0.33 | NA |
|  |  |  | Methanobrevibacter smithii | 50 | 2.32 | NA |
|  |  |  | Methanosphaera stadtmanae | 30 | 1.37 | NA |
|  |  |  | Mycoplasma hominis | 100 | 1.83 | NA |
|  |  |  | Odoribacter splanchnicus | 50 | 5.70 | NA |
|  |  |  | Oxalobacter formigenes | 30 | 1.77 | NA |
|  |  |  | Paraprevotella xylaniphila | 20 | 2.13 | NA |
|  |  |  | Parvimonas micra | 10 | 0.43 | NA |
|  |  |  | Peptoniphilus harei | 10 | 0.45 | NA |
|  |  |  | Phascolarctobacterium succinatutens | 20 | 1.22 | NA |
|  |  |  | Porphyromonas asaccharolytica | 5 | 0.28 | NA |
|  |  |  | Prevotella melaninogenica | 20 | 0.73 | NA |
|  |  |  | Roseburia intestinalis | 10 | 1.17 | NA |
|  |  |  | Rothia dentocariosa | 15 | 0.98 | 79.17 |
|  |  |  | Rothia mucilaginosa | 20 | 1.22 | 79.17 |
|  |  |  | Ruminococcus gnavus | 5 | 0.48 | NA |
|  |  |  | Staphylococcus epidermidis | 15 | 0.82 | NA |
|  |  |  | Streptococcus agalactiae | 5 | 0.24 | NA |
|  |  |  | Streptococcus anginosus | 15 | 0.80 | 80.41 |
|  |  |  | Streptococcus australis | 30 | 1.57 | 85.17 |
|  |  |  | Streptococcus cristatus | 20 | 1.12 | 81.43 |
|  |  |  | Streptococcus mutans | 20 | 1.07 | NA |
|  |  |  | Streptococcus parasanguinis | 30 | 1.68 | 85.17 |
|  |  |  | Veillonella dispar | 5 | 0.27 | 86.84 |
|  |  |  | Veillonella parvula | 10 | 0.55 | 86.84 |
|  |  |  | Weissella cibaria | 15 | 0.92 | NA |

^a^ Sequence depth denotes the target sequencing depth used for read simulation. ^b^ Relative abundance was calculated from the simulated read output assigned to each reference genome. ^c^ Max genomic relatedness indicates the highest pairwise average nucleotide identity (ANI) between each species reference genome and the other species reference genomes in the dataset, as calculated using FastANI [2]. A few estimates slightly below 80% were returned under the default settings; these values should be interpreted with caution because FastANI is primarily validated within the 80-100% ANI range. ^d^ NA indicates that FastANI did not return a valid pairwise ANI estimate for the focal genome after excluding self-comparisons, owing to insufficient homologous fragment mappings or genomic similarity far below 80%.

**Table S2. Composition details of the three experimental mock-community datasets**

| Dataset ^a^ | Count of Reads | Species | Species-level relative  abundance ^b^ (%) | Max genomic  relatedness ^c^ (%) |
| --- | --- | --- | --- | --- |
| SRR13128012  (SRX9569059) | 2480208 | Faecalibacterium prausnitzii | 14.82 | NA ^d^ |
|  |  | Veillonella rogosae | 20.01 | NA |
|  |  | Roseburia hominis | 12.47 | NA |
|  |  | Bacteroides fragilis | 8.36 | NA |
|  |  | Prevotella corporis | 6.28 | NA |
|  |  | Bifidobacterium adolescentis | 8.86 | NA |
|  |  | Fusobacterium nucleatum | 7.56 | NA |
| SRR13128013  (SRX9569058) | 2770833 | Lactobacillus fermentum | 9.71 | NA |
|  |  | Clostridioides difficile | 1.10 | NA |
|  |  | Akkermansia muciniphila | 1.62 | NA |
|  |  | Methanobrevibacter smithii | 0.17 | NA |
|  |  | Salmonella enterica | 0.0065 | 82.33 |
|  |  | Enterococcus faecalis | 0.0011 | NA |
|  |  | Clostridium perfringens | 0.00009 | NA |
| SRR13128014  (SRX9569057) | 1978852 | Escherichia coli (JM109) | 1.83 | 99.53 |
|  |  | Escherichia coli (B-3008) | 1.82 | 98.96 |
|  |  | Escherichia coli (B-2207) | 1.65 | 99.33 |
|  |  | Escherichia coli (B-766) | 1.66 | 98.48 |
|  |  | Escherichia coli (B-1109) | 1.77 | 99.53 |
|  |  | Candida albicans | 0.16 | NA |
|  |  | Saccharomyces cerevisiae | 0.16 | NA |

^a^ SRR identifiers denote the NCBI Sequence Read Archive run accessions, with the corresponding experiment accessions provided in parentheses. ^b^ Species-level relative abundance values were reproduced from LRBinner [3]. ^c^ Max genomic relatedness indicates the highest pairwise average nucleotide identity (ANI) between each species reference genome and the other species reference genomes in the dataset, as calculated using FastANI [2]. ^d^ NA indicates that FastANI did not return a valid pairwise ANI estimate for the focal genome after excluding self-comparisons, owing to insufficient homologous fragment mappings or genomic similarity far below 80%.

**Table S3. Composition details of the strain-level resolution dataset**

| Dataset | Count of Reads | Total Size | Strain ID | Species | Sequence depth (x) ^a^ | Species-level relative abundance (%) ^a^ | Genomic  relatedness (%) ^b^ |
| --- | --- | --- | --- | --- | --- | --- | --- |
| NWC2 | 763,335 | 4.7GB | S.thermophilus  NWC_2_1 | Streptococcus  thermophilus | 833 | 45.4 | 74.77 |
|  |  |  | L. delbrueckii  NWC_2_2 | Lactobacillus  delbrueckii | 273 | 24.5 | 76.88 |
|  |  |  | L. helveticus  NWC_2_3 | Lactobacillus  helveticus | ~30 | 25.6 combined | 98.94 |
|  |  |  | L. helveticus  NWC_2_4 | Lactobacillus  helveticus | ~60 | 25.6 combined | 98.94 |

^a^ Sequence depth and species-level relative abundance values were obtained from the original NWC2 study [4]. The reported PacBio sequencing depths were 833× for S. thermophilus NWC_2_1 and 273× for L. delbrueckii NWC_2_2. The two L. helveticus strains, NWC_2_3 and NWC_2_4, were represented by approximately 30× and 60× coverage groups, respectively, based on strain-specific genomic regions; regions shared by the two strains exhibited approximately 90× coverage. Species-level relative abundances were derived from 16S rRNA profiling. The value of 25.6% represents the combined abundance of NWC_2_3 and NWC_2_4 rather than the abundance of each strain individually. ^b^ Genomic relatedness denotes the pairwise average nucleotide identity of each strain genome to another strain genome in the dataset, as calculated using FastANI [2]. To facilitate comparisons among relatively divergent genomes, the default --fragLen and --minFraction values were adjusted from 3,000 bp and 0.20 to 1,000 bp and 0.05, respectively. The --fragLen parameter specifies the length of genome fragments used for sequence mapping, whereas --minFraction specifies the minimum fraction of genome fragments that must be successfully mapped for an ANI estimate to be reported. These relaxed parameter settings enabled FastANI to report ANI values below 80%. Because FastANI is primarily validated within the 80-100% ANI range, the estimates below 80% obtained under these relaxed settings should be interpreted with caution.

**Table S4. Performance of DecBin with different k-mer sizes on three simulated datasets**

| Datasets | k | Number of bins | ACC (%) | Feature storage size (GB) ^a^ |
| --- | --- | --- | --- | --- |
| sim10 | 3 | 9 | 81.04 | 0.11 |
|  | 4 | **10** | **85.06** | 0.47 |
|  | 5 | 12 | 82.21 | 1.78 |
|  | 6 | 11 | 82.93 | 7.23 |
| sim30 | 3 | 21 | 69.83 | 0.15 |
|  | 4 | **28** | 68.78 | 0.66 |
|  | 5 | 26 | **70.55** | 2.47 |
|  | 6 | 24 | 67.86 | 10.04 |
| sim70 | 3 | 49 | 48.00 | 0.20 |
|  | 4 | 58 | 48.98 | 0.87 |
|  | 5 | 54 | 48.32 | 3.27 |
|  | 6 | **61** | **49.74** | 13.30 |

^a^ Feature storage size denotes the total disk space occupied by the k-mer compositional features generated for all reads in the corresponding dataset. Bold values indicate the number of bins closest to the expected species number and the highest ACC for each dataset.

**Table S5. Runtime and peak memory usage of MetaBCC-LR, LRBinner, and DecBin on three simulated datasets**

| Datasets | Method | Time cost (min.) | Peak memory (GB) |
| --- | --- | --- | --- |
| sim10 | MetaBCC-LR | 4.42 | 4.27 |
|  | LRBinner | 13.21 | 4.74 |
|  | DecBin | 22.38 | 5.88 |
| sim30 | MetaBCC-LR | 6.58 | 4.27 |
|  | LRBinner | 16.40 | 4.91 |
|  | DecBin | 27.76 | 6.81 |
| Sim70 | MetaBCC-LR | 7.55 | 4.27 |
|  | LRBinner | 21.02 | 5.12 |
|  | DecBin | 35.01 | 7.52 |

**Table S6. Cluster-number estimation by random search and Bayesian optimization on three simulated datasets**

| Dataset | Search strategy | Estimated number of clusters | Expected number of species |
| --- | --- | --- | --- |
| sim10 | Random search | 10±2 | 10 |
|  | Bayesian optimization | 10±2 |  |
| sim30 | Random search | 23±2 | 30 |
|  | Bayesian optimization | 28±2 |  |
| sim70 | Random search | 50±3 | 70 |
|  | Bayesian optimization | 58±4 |  |

Random search and Bayesian optimization used the same search range, number of candidate evaluations, and DBI objective.

**Table S7. Completeness, contamination and purity of high- and medium-quality MAGs recovered by DecBin, LRBinner, and MetaBCC-LR from three experimental mock-community datasets.**

| Datasets | DecBin | | | LRBinner | | | MetaBCC-LR | | | |
| --- | --- | --- | --- | --- | --- | --- | --- | --- | --- | --- |
|  | Completeness | Contamination | Purity | Completeness | Contamination | Purity | Completeness | Contamination | Purity | (%) |
| SRR131  28012 | 100.00 | 5.26 | 96.86 | 100.00 | 0.24 | 98.78 | 99.98 | 1.12 | 97.15 |  |
|  | 100.00 | 8.98 | 99.24 | 99.98 | 0.56 | 97.10 | 90.60 | 0.06 | 99.99 |  |
|  | 99.51 | 5.32 | 96.99 | 99.97 | 0.21 | 99.72 | 82.40 | 1.41 | 99.74 |  |
|  | 99.32 | 1.36 | 96.76 | 99.97 | 0.01 | 99.24 |  |  |  |  |
|  | 99.18 | 1.00 | 99.81 | 99.97 | 0.17 | 99.05 |  |  |  |  |
|  | 99.08 | 0.24 | 99.88 | 98.95 | 1.73 | 97.59 |  |  |  |  |
|  | 97.96 | 0.00 | 97.86 |  |  |  |  |  |  |  |
|  | 73.40 | 2.43 | 97.61 |  |  |  |  |  |  |  |
| SRR131  28013 | 100.00 | 0.68 | 99.48 | 100.00 | 1.36 | 98.25 | 100.00 | 7.46 | 83.17 |  |
|  | 100.00 | 0.13 | 99.70 | 99.44 | 0.35 | 97.88 | 100.00 | 0.25 | 99.72 |  |
|  | 99.51 | 1.86 | 97.87 | 97.96 | 0.00 | 99.27 | 100.00 | 0.16 | 99.87 |  |
|  | 99.28 | 0.80 | 98.06 |  |  |  | 99.97 | 0.18 | 99.99 |  |
|  | 99.18 | 0.55 | 99.96 |  |  |  |  |  |  |  |
|  | 97.96 | 0.00 | 99.02 |  |  |  |  |  |  |  |
| SRR131  28014 | 100.00 | 0.08 | 99.07 | 100.00 | 0.35 | 96.22 | 100.00 | 0.23 | 99.99 |  |
|  | 100.00 | 4.25 | 99.88 | 99.98 | 0.20 | 98.59 | 99.97 | 0.06 | 99.85 |  |
|  | 100.00 | 1.14 | 99.75 | 99.97 | 0.19 | 99.52 | 99.96 | 0.07 | 99.99 |  |
|  | 99.51 | 2.45 | 99.06 | 99.96 | 0.06 | 99.21 | 99.95 | 0.20 | 99.73 |  |
|  | 99.44 | 4.62 | 97.17 | 71.92 | 6.71 | 46.74 | 98.96 | 0.00 | 99.55 |  |
|  | 99.18 | 1.09 | 97.62 |  |  |  | 66.90 | 0.27 | 99.82 |  |
|  | 97.96 | 0.00 | 98.91 |  |  |  |  |  |  |  |
|  | 96.41 | 8.16 | 99.06 |  |  |  |  |  |  |  |
|  | 90.15 | 0.45 | 99.51 |  |  |  |  |  |  |  |
|  | 88.78 | 0.00 | 99.79 |  |  |  |  |  |  |  |
|  | 70.93 | 1.01 | 99.48 |  |  |  |  |  |  |  |
|  | 67.97 | 0.14 | 99.66 |  |  |  |  |  |  |  |

**Figure S1. Single-copy marker-gene detection rates on the sim10 dataset.**

**Figure S2. Single-copy marker-gene detection rates on the sim30 dataset.**

**Figure S3. Single-copy marker-gene detection rates on the sim70 dataset.**

**Reference**

[1]	D. E. Wood, J. Lu, and B. Langmead, Improved metagenomic analysis with Kraken 2, *Genome Biology,* 2019, 20 (1): 257.

[2]	C. Jain, L. M. Rodriguez-R, A. M. Phillippy, K. T. Konstantinidis, and S. Aluru, High throughput ANI analysis of 90K prokaryotic genomes reveals clear species boundaries, *Nature Communications,* 2018, 9 (1): 5114.

[3]	A. Wickramarachchi, and Y. Lin, Binning long reads in metagenomics datasets using composition and coverage information, *Algorithms for Molecular Biology,* 2022, 17 (1): 14.

[4]	V. Somerville, S. Lutz, M. Schmid, D. Frei, A. Moser, S. Irmler *et al.*, Long-read based de novo assembly of low-complexity metagenome samples results in finished genomes and reveals insights into strain diversity and an active phage system, *BMC Microbiology,* 2019, 19 (1): 143.
